# Assessment of the biological potential of diaryltriazene-derived triazene compounds

**DOI:** 10.1038/s41598-021-81823-2

**Published:** 2021-01-28

**Authors:** Patricia de Maria Silva Figueirêdo, José Costa Sampaio Filho, Alzirene de Jesus Sales Sodré, José Ribamar de Castro Júnior, Ingrid Santos Gonçalves, Rodrigo Vieira Blasques, Rodrigo S. Correa, Benedicto Augusto Vieira Lima, Larissa dos Anjos Marques, Denise Fernandes Coutinho, Ana Paula Silva de Azevedo dos Santos, Tássio Rômulo Silva Araújo Luz, Rita de Cassia Mendonça de Miranda, Julliana Ribeiro Alves dos Santos, Antonio Carlos Doriguetto, María Isabel Pividori, Manfredo Hörner, Paulo Cesar Mendes Villis

**Affiliations:** 1 Laboratório de Microbiologia Clínica, Federal University of Maranhão (UFMA), São Luís, MA 65.080-040 Brazil; 2 Electrochemistry and Biotechnology Laboratory (EBL), University of CEUMA (UNICEUMA), São Luís, MA 65.065-470 Brazil; 3 Department of Nature Sciences, Mathematics, and Education, Federal University of São Carlos, Araras, SP 13.600-970 Brazil; 4 Instituto de Ciências Exatas e Biológicas (ICEB), Federal University of Ouro Preto (UFOP), Ouro Preto, MG 35.400-000 Brazil; 5 Licenciatura em Ciências Naturais, Federal University of Maranhão (UFMA), Grajaú, MA 65.940-000 Brazil; 6 Laboratório de Farmacognosia, Federal University of Maranhão (UFMA), São Luís, MA 65.080-040 Brazil; 7 Laboratório de Imunologia Aplicada ao Câncer (LIAC), Federal University of Maranhão (UFMA), São Luís, MA 65.080-040 Brazil; 8 Laboratory of Environmental Microbiology, University of CEUMA (UNICEUMA), São Luís, MA 65.065-470 Brazil; 9 Institute of Chemistry, Federal University of Alfenas (UNIFAL), Alfenas, MG 37.130-001 Brazil; 10 Grup de Sensors i Biosensors, Universitat Autònoma de Barcelona, 08193 Bellaterra, Spain; 11 Department of Chemistry, Federal University of Santa Maria (UFSM), Santa Maria, RS 97.110-900 Brazil

**Keywords:** Microbiology, Chemistry, Drug discovery

## Abstract

In the present study, novel, 1,3-diaryltriazene-derived triazene compounds were synthesized and tested. Triazenes are versatile and belong to a group of alkylating agents with interesting physicochemical properties and proven biological activities. This study describes the synthesis, molecular and crystalline structure, biological activity evaluation, and antifungal and antimicrobial potentials of 1,3-*bis*(X-methoxy-Y-nitrophenyl)triazenes [X = 2 and 5; Y = 4 and 5]. The antimicrobial and antifungal activities of the compounds were tested by evaluating the sensitivity of bacteria (American Type Culture Collection, ATCC) and clinical isolates to their solutions using standardized microbiological assays, cytotoxicity evaluation, and ecotoxicity tests. The antimicrobial potentials of triazenes were determined according to their minimum inhibitory concentrations (MICs); these compounds were active against gram-positive and gram-negative bacteria, with low MIC values. The most surprising result was obtained for **T3** having the effective MIC of 9.937 µg/mL and antifungal activity against *Candida albicans* ATCC 90028, *C. parapsilosis* ATCC 22019, and *C. tropicallis* IC. To the best of our knowledge, this study is the first to report promising activities of triazene compounds against yeast and filamentous fungi. The results showed the potential utility of triazenes as agents affecting selected resistant bacterial and fungal strains.

## Introduction

The extensive use of antimicrobial drugs has resulted in antibiotic resistance becoming a serious health issue^1^ with consequences affecting people around the world^2^. There is an urgent need to develop new effective antibacterial agents that will circumvent the emerging resistance^3^. To this end, new triazene compounds based on substituted 1,3-diaryltriazene were synthesized in the current study.

**Figure 1:**
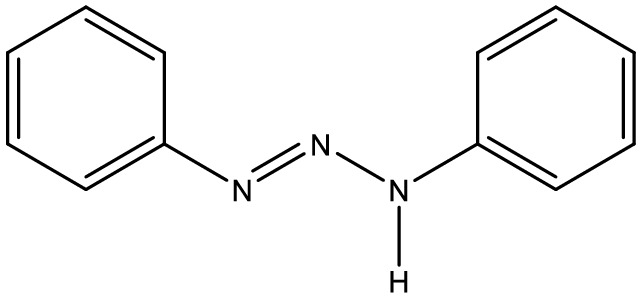
Structure of 1,3-*bis*(phenyl)triazene.

Triazenes are azo compounds^4^, isoelectronic^5^, and characterized by a diazoamino group (–N=N–N)^6–8^ containing three consecutive nitrogen atoms^9^. The synthesis of the first triazene dates from 1859^10^ to a study on diazonium salt preparation that described the first symmetric triazene compound, diaryltriazene [1,3-*bis*(phenyl)triazene; Fig. 1].

The triazene class of compounds is large and variable^11,12^ with the possibility of numerous biological applications, however, despite its relevance, few studies have been reported in the literature on the biological activity of triazene compounds. These compounds are promising for a broad range of applications. In industry, they can be used as chemical and biological reagents^13,14^ and in organic synthesis^15–17^. The pharmaceutical industry has invested in studies on action against microorganisms^18,19^, functioning as appetite reducers^20,21^, anticancer agents^22–26^ such as melanoma-specific^25^ and primary brain tumors^28^ therapeutics, fluorescence sensing agents^29^, mutagenic factors^12^, and having diabetic, anti-inflammatory, and antioxidant^27^ activities; triazenes also exhibit biological and environmental activities^6,30–33^.

New diaryltriazene-derived compounds have been synthesized, and their antimicrobial properties have also been evaluated^34^. Previously^19^, the antimicrobial activities of diaryltriazenes, including antifungal activity of novel hydroxytriazenes (*Candida albicans, Cryptococcus neoformans, Sporotrichum schenckii, Trichophyton mantagrophytes*, and *Aspergillus fumigatus*)^20^, anorexics, and anticancer activity^34^ have been explored. In these studies, the alkyltriazenes’ mechanism of action was generally linked to the formation of reactive diazonium species capable of DNA alkylation^12^.

Hörner et al.^35^ reported the in vitro antibacterial activities of newly synthesized diaryltriazenes against standard bacteria (American Type Culture Collection, ATCC) and clinical isolates including multidrug-resistant (MDR) bacteria, and acute toxicity against *Artemia salina*. Initial experiments showed that these triazene compounds have biological activity against both methicillin-resistant *Staphylococcus aureus* (MRSA) and *Mycobacterium smegmatis* strains, with MIC values of 0.02 and 0.03 mg/mL, respectively^27,36^.

Hörner et al*.*^37^ evaluated the activity of 15 triazenide compounds including salts of vanadium and potassium hydroxytriazene complexes against various bacteria involving extended-spectrum β-lactamase (ESBL) strains. The compounds were active against multidrug-resistant bacteria including ESBL-, metallo-β-lactamases (MBL)-, and adenosine 3,5-cyclic monophosphate (AmpC) gene-producing strains. This evidence has had a major impact because these mechanisms of bacterial resistance are among the most prevalent. Several triazenes with potential antimicrobial activity against MRSA and *M. Smegmatis* have been identified^34^.

Antibiotic resistance should be assessed from ecological and environmental perspectives^2^. Environmental contamination involved in the new compounds discovery and use is of global concern^38^. There is a need for rapid synthesis of small molecules with increased efficiency and minor side effects compared to existing ones, in a simple, effective, and environmentally friendly way^39^; it is necessary to report new agents that are effective against microorganisms to balance demand; triazene compounds promise such potential. Thus, 2-phenyl-1,3-benzodioxole and 2-phenyl-1,3-benzodioxol-4-ol derivatives were synthesized using the green chemistry approach^40^, and were evaluated for anticancer and antibacterial effects^41^.

The active moiety of triazene compounds is the triazenyl group^12^ that confers their chemical, physical, and antitumor properties and the ability to form hydrogen bonds. Triazene compounds^12^ including dacarbazines (DTIC; BTIC)^18,42^ and its derivatives^18^, temozolomide (TMZ)^43^, and mitozolomide^44^ are alkylating agents. However, only the first two agents are presently used in clinical practice; mitozolomide remains an experimental antitumor drug due to its low tolerability. Table 1 shows some diaryltriazene derivatives with biological activity. Another triazene, *Berenil*^23,45^, a photostable DNA-binding ligand, has been the drug of choice for treating animal trypanosomiasis^46^; 3,3-dialkyl-1-aryltriazene gives it potential antimalarial activity^47^.

**Table 1:** Diaryltriazene derivatives with biological activities.

Compounds	Business name	Properties
5-(3,3-Dimethyl-1-triazenil)imidazol-4-carboxamide	Dacarbazine (DTIC)	antineoplastic
5-[3,3-*Bis(*2-chloroethyl-1-triazenil]imidazol-4-carboxamide	Dacarbazine (BTIC)	antineoplastic
3-Methyl-4-oxo-3,4-dihidroimidazol[5,1-*d*][1,2,3,5]tetrazine-8-carboxiamide	Termozolomide (TEM)	antineoplastic
1,3-*Bis*(4′-amidinophenyl)triazene *N*-acetylglycine	Berenil	antitripanosomal

Studies of triazenes antitumor activity have been reported. Cytarska et al.^48^ investigated the antiproliferative activity of nine triazene salts against human cancer cell lines. The results showed that the compounds (triazene salts) had very strong activity against *Burkitt lymphoma* DAUDI and human colon adenocarcinoma HT-29 cells, with IC-50 values of 4.91 µg/mL and 5.59 µg/mL, respectively. Recently, Seck et al.^36^ reported new triazene compounds and evaluated their antimicrobial properties. All strains were sensitive, with the best MIC of 0.28 μM being observed against *Escherichia coli*, satisfactory against *Pseudomonas aeruginosa*, and 0.64 μM against *Enterococcus faecalis.*

The work presented here describes the synthesis and molecular and crystalline characterization of triazene compounds derived from diaryltriazenes. Triazene compounds have promising biological activities and can be used as anti-tumor agents by reducing the size of neoplastic cells, and also act as effective antibacterial agents that can be used as an alternative to control emerging microbial resistance. This is the first report of the activity of triazenes against yeasts and filamentous fungi. This finding is of significance due to the prevalence of resistance among microorganisms. These new triazene compounds have been evaluated for biological and antimicrobial activities of clinical interest using standardized microbiological assays.

## Experimental

### Materials, instrumentation, and methods

All chemicals used were of analytical reagent grade (Sigma-Aldrich, São Paulo, SP, Brazil). All solvents used in this study were used without purification (Sigma-Aldrich, São Paulo, SP, Brazil).

Elemental analyses were carried out in triplicate on a Leco Corporation Model TruSpec Micro CHNS/O Analyzer (State University of Campinas, UNICAMP, Campinas, SP, Brazil). The melting point was determined using a Mel-Temp II (Federal University of Alfenas (UNIFAL), Alfenas, MG, Brazil). Infrared spectra were recorded using a Shimadzu IR Prestige-21 FTIR spectrometer (Federal University of Alfenas (UNIFAL), Alfenas, MG, Brazil). Hydrogen nuclear magnetic resonance (^1^H NMR) spectra were obtained on a Bruker DPX-300 spectrometer (Federal University of Santa Maria, Santa Maria, RS, Brazil). ^13^C high-power proton decoupling (HPDEC) magic-angle spinning (MAS) NMR spectra were obtained at 298 K on a Bruker AC 300P spectrometer (State University of Campinas, Campinas, SP, Brazil) operating at 75.43 MHz for ^13^C. Tetramethylsilane (TMS) [Si(CH_3_)_4_] was used as an external reference for chemical shifts. Ultraviolet–visible spectroscopy (UV–Vis) analysis was performed using a *spectrophotometer (Spectroquant Prove 600,* University of CEUMA (UNICEUMA), São Luís, MA, Brazil*).* A GC 1000 gas chromatograph (GC) with a flame ionization detector (FID) (Ciola Gregori Ltda., São Paulo, Brazil) equipped with a splitless injector inlet liner interfaced to a PC with a DANI DS 1000 integrator (Dani Strumentazione Analitica, Monza, Italy) and IQ3 software for data acquisition were used. *S*tructural analysis by single-crystal X-ray diffraction was performed using a Bruker APEX II-CCD area-detector diffractometer and graphite monochromatized Mo-K_α_ radiation (λ = 0.71073 Å) (Federal University of Santa Maria (UFSM), Santa Maria, RS, Brazil).

Compounds **1**, **2**, and **3** were synthesized as described previously^49^ (see also Supplementary Material: Supplemental methods).

### Tested microorganisms

Standard ATCC microorganisms, namely *Staphylococcus aureus* ATCC 25923, *Staphylococcus hemolyticus* IC 13084879, *Streptococcus pyogenes* ATCC 19615, *Pseudomonas aeruginosa* ATCC 27853, *Escherichia coli* ATCC 25922, *Salmonella* Enteritidis ATCC 13076, *Proteus mirabilis* 7002, and *Acinetobacter baumannii* ATCC 19606 were used for the tests. Fungal strains of *Candida albicans* ATCC 90028, *C. parapsilosis* ATCC 22019, and *C. krusei* ATCC 6258 were used. Also, *Fusarium oxysporum* and *Penicillium janthinellum* strains of filamentous fungi were obtained from the Federal University of Maranhão (UFMA).

The samples were kept frozen until use. The microorganisms were reactivated from their original cultures and kept in liquid BHI (Brain Heart Infusion) at 37 °C for 24 h. Subsequently, the samples were grown on nutrient agar plates at 37 °C for 24 h for bacteria and 48 h for fungi for later identification in specific media.

All tests for antimicrobial activity were performed on Mueller Hinton Agar or Broth Mueller Hinton.

### Agar diffusion antimicrobial activity test

Each triazene (20 µL) was separately applied to sterile paper discs. A disk impregnated with each triazene was placed on the bacterium plate, with a positive (chloramphenicol)^50^ and two negative (isopropyl alcohol and ethanol from which triazene compounds were obtained) controls. The plates were placed in an incubator at 37 °C for 24 h and the results were observed^51^.

### Determination of minimum inhibitory concentration (MIC)

The MIC of compounds 1, 2, and 3 was determined using the microdilution technique described by the Clinical and Laboratory Standards Institute^52^. To a sterile 96-well microplate, 150 µL of Mueller Hinton broth and 150 µL of the test material were added, followed by serial dilutions up to 1/64. As a positive control, 20 µL of the antimicrobial (chloramphenicol 0.02 µg/mL or nystatin 100,000 IU) specific for each microorganism was used. As the negative control, 150 µL of the diluent or base was added to assess antimicrobial activity. Finally, 5 µL of the microbial suspension prepared in 0.9% saline was added, according to the McFarland 0,5 scale (1.5 × 10^8^ CFU/mL). The microplate was incubated for 24 to 48 h in an incubator at 37 °C and resazurin was added to determine microbial growth^53^. The MIC for yeast was determined by a modified broth microdilution method^54^.

### Antifungal activity against filamentous fungi using the poisoned food method

Poisoned food evaluation is a variant of the agar dilution method^55^. The main difference is the inoculation by microorganisms is in only one point of the plate to measure radial growth. Thus, this technique was applied to evaluate antimicrobial activity against filamentous fungi. The compound was incorporated into the molten culture medium before pouring into Petri dishes. The target filamentous fungus was inoculated in the center of the plate and incubated for growth. After growth, the diameter was measured and compared to that on the control plates^56^.

### Determination of antibiotic potency

Mueller Hinton broth (150 µL) was transferred into 96-well plastic plates. Serial dilutions were prepared by adding an equal volume of chloramphenicol (initial concentration 20 µg/mL), and after mixing, the same volume was transferred to the next well. Bacterial colonies that showed the best MIC results were isolated in the appropriate medium and those with similar morphological patterns were selected. The colonies were suspended in 0.9% saline solution so that the turbidity of the colonies was similar to the standard MacFarland scale of 0.5. Bacterial suspensions (5 µL) were inoculated into each well^57^.

To determine the potency of the test triazene compounds, a comparison was made between their MIC results and those of the known antibiotics, according to Eq. 1

1 $$P = \left( {MICt / MICc} \right) \times 100$$

where MICt: MIC of triazene compound, MICc: MIC of chloramphenicol, P: Potency.

### Assessment of mucous membrane irritation potential

PBS was prepared by dissolving pre-determined amounts of monobasic potassium phosphate, dibasic potassium phosphate, and sodium chloride in 1.0 L of distilled water, and pH was adjusted if necessary to 7.2 ± 0.2. 2 mL of defibrinated sheep blood was placed into a screwable tube with a lid and centrifuged at 3000 rpm for 15 min. After discarding the supernatant, the red blood cells were subjected to three consecutive washes with saline phosphate buffer to remove the remaining plasma. Serial dilutions of the triazene were carried out in phosphate buffer saline, with a mixture of 975 µL of buffer and the same volume of the triazenes and transferred to other tubes diluting the triazene. Subsequently, discarding the final excess volume. The red blood cell suspension (25 µL) was added to the mixture (completing the volume to 1000 µL). At the end of this step, the suspension formed by mixing the product with the buffer and the red cells was incubated at room temperature for 10 min followed by centrifugation at 10,000 rpm for one min. The supernatant was transferred to a 1 cm^3^ cuvette, and the absorbance was measured using a UV spectrophotometer at wavelengths of 540 and 575 nm. The control suspension was composed of phosphate buffer saline and the test product; the blank was PBS only so that it minimized the interference in the reading of the data. The results were statistically analyzed using Pearson and Spearman coefficients to verify the statistical validity of the irritability classification assigned to the triazenes^58^.

An in vitro lesion assay was performed in sheep red blood cells (see also Supplementary Material), according to the method described by Alves et al.^59^.

The triazene compounds were characterized by their irritability potential based on the parameters shown in Table S1 (see Table S1 of the Supplementary Material).

### MTT cytotoxicity test

Cytotoxicity of triazenes was examined by determining the viability of RAW 264.7 (murine macrophages), breast adenocarcinoma (MCF-7), prostate carcinoma (DU-145), HeLa (uterine carcinoma), and human lung fibroblast (GM) cells after treatment with the tested compounds and evaluated by the colorimetric assay based on the reduction of tetrazolium salt, 3-(4,5-dimethyl-2-thiazolyl bromide)-2,5-diphenyl-2H tetrazolium (MTT; Sigma-Aldrich, St. Louis, MO, USA)^60^. The cells (1 × 10^6^ cell/mL) were grown in 96-well plates and incubated for 1 h in RPMI medium at 37 °C in 5% CO_2_ and then treated with the MIC of the triazenes for 24 h. After incubation, 10 μL of MTT (5 mg/mL) was added to the cultures. After 3 h of incubation, 100 μL of 10% SDS was added. The plates were incubated overnight at room temperature in the dark, and optical density (OD) was measured at 540 nm to determine cell viability and was compared with the negative (without treatment) and positive (Triton X-100-treated cells) controls.

The prostate (DU145), cervix (HeLa), and breast cancer (MCF7) cell lines were obtained from the Bank of Cells of Rio de Janeiro (NCE/UFRJ; http://bcrj.org.br/). The cell line of normal human lung fibroblasts (GM07492A) was used as a control per the recommendations of the Coriell Institute for Medical Research (https://www.coriell.org).

### Ecotoxicity test using *Danio rerio* (zebrafish)

The ecotoxicological test was performed following the methodology of the Brazilian Association of Technical Standards (ABNT), Brazilian Standard (NBR Nº 15088, of 13.12.2016)^61^ and using adult *Danio rerio* (3.0 to 4.0 cm in length). The required quantities of each product and extract were calculated to obtain the MIC in 1000 mL of water. Triplicate dilutions were made in acrylic aquaria and groups of four fish were added to each. Fish behavior was observed for 15 min, 24 h, and 48 h to record how many died. As a negative control, 0.1% DMSO in water was used. The results were expressed qualitatively as "Toxic" or "Non-Toxic" based on the exposure time.

### Animal assay

Animal experiments were performed according to the ARRIVE guidelines (Animal Research: Reporting of In Vivo Experiments; http://www.nc3rs.org.uk/arrive-guidelines)^62^, and as required by the Ethics Committee on Animal Use of the Federal University of Maranhão (UFMA), Maranhão, Brazil (Record: 23115.009327/ 2017-10). All experiments with live vertebrates and/or higher invertebrates were performed in strict accordance with relevant guidelines and with the Brazilian Federal Law 11,794, 10.08.2008, and National Animal Experimentation Control Council (CONCEA) Federal Normative Resolution 34, 07.27.2017, that established procedures for the scientific use of animals.

### Statistical analyses

Results were expressed as the mean ± standard error and analyzed using analysis of variance (one-way ANOVA) for multiple comparisons, followed by Tukey’s test. All statistical analyses were conducted at a 95% (p < 0.05) significance level using GraphPad Prism 8.0 software.

## Results and discussion

### Chemistry

**Scheme 1:**
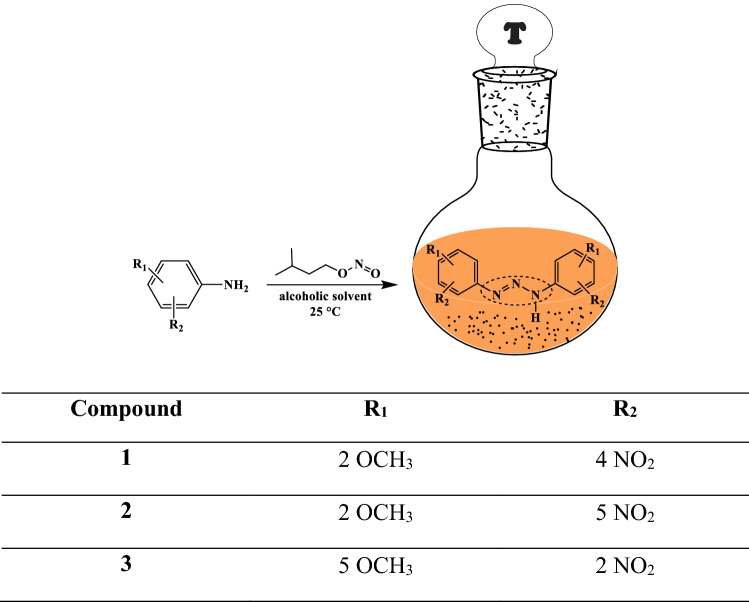
Synthesis of triazene compounds **1**, **2**, and** 3**.

**Figure 2:**
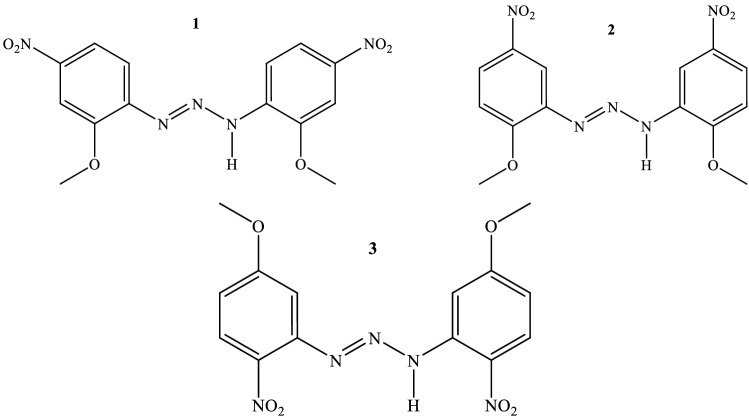
The structures of 1,3-diaryltriazenes **(1)** 1,3-*bis*(2-methoxy-4-nitrophenyl)triazene, **(2)** 1,3-*bis*(2-methoxy-5-nitrophenyl)triazene, and **(3)** 1,3-*bis*(5-methoxy-2-nitrophenyl)triazene.

1,3-Diaryltriazenes **1**, **2**, and** 3** were prepared by treating the appropriately substituted anilines with isoamyl nitrite in an alcoholic medium at room temperature using the method of Vernin et al.^49^ according to Scheme 1; their structures are shown in Fig. 2.

The synthesis and structural characterization used in this study are summarized in Supplementary Material Figs. S1, S2, and S3.

### X-ray crystallography

The crystal structure of compound **1** shows the expected *trans* stereochemistry of the N=N double bond suggesting that its molecular structure corresponds to the commonly observed *trans* stereochemistry of the N11=N12 double bond^63^, reflecting typical characteristics of free diaryltriazene *π* electron delocalization on the diazoamino moiety terminally substituted aryls of the triazene group. The N11=N12 bond [1.2974(18) Å] is longer than the characteristic double bond value (1.236 Å)^63^, whereas the N12-N13 bond [1.3158(18) Å)] is shorter than the characteristic single bond value (1.404 Å)^63^. Conversely, the C1-N11 [1.418(2) Å] and C7-N13 [1.4011(19) Å] bonds are shorter than the characteristic N-C_aryl_ single bonds (secondary amines, *R*_2_NH, *R* = C*sp*^2^; 1.452 Å)^64^. These values agree with those observed for similar compounds such as 1,3-di(2-methoxyphenyl)triazene [N = N = 1.270 (2) Å and N–N = 1.323(2) Å]^65^ and 1,3-*bis*(4-nitrophenyl)triazene [N = N = 1.263(2) Å and N–N = 1.340(2) Å]^66^. The N = N–N triad [N11-N12-N13 = 112.12(13)°] bond angle is very close to that found in 1,3-*di*(2-methoxyphenyl)triazene [112.47°], and 1,3-*bis*(4-nitrophenyl)triazene [112.32(15)°]^66^.

**Figure 3:**
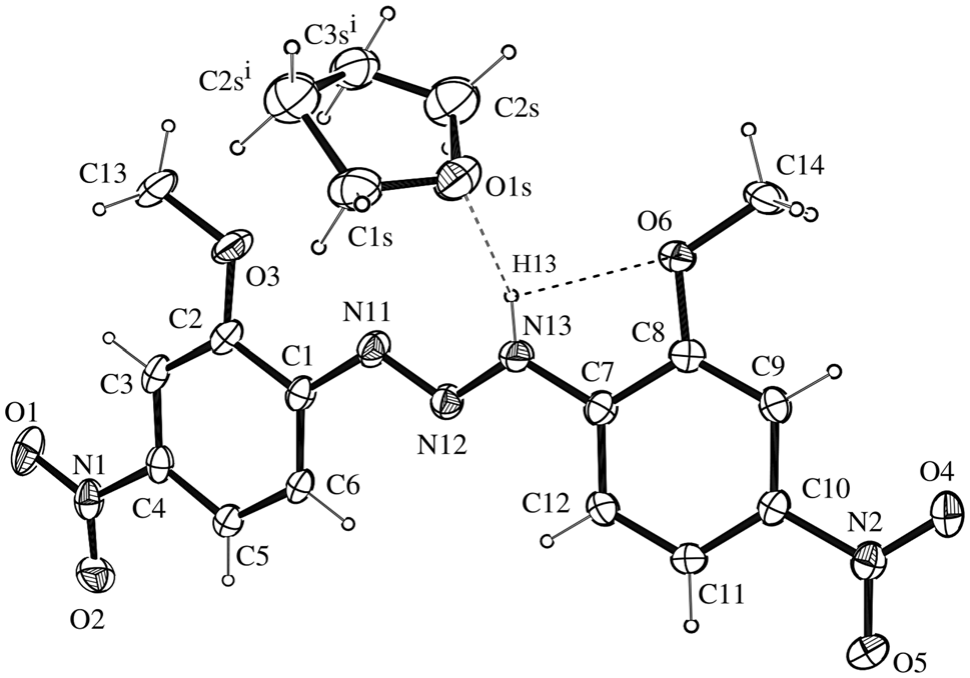
Molecular structure of **1,** with the atom-numbering scheme. Displacement ellipsoids are drawn at the 50% probability and H atoms have arbitrary radii. The intermolecular N13-H···O1s hydrogen bonds between **1** and THF is shown as a dashed line. Symmetry code (i) − *x* + *1, − y, − *z. The intramolecular N13 − H13⋅⋅⋅O6 interaction is shown as a dashed line. Cambridge Crystallographic Data Centre (CCDC N. 661902).

The diazoamino moiety of 1,3-disubstituted triazenes and strategically substituted terminal aryl substituents provide intermolecular interactions through hydrogen bonds, with polarizable and electronegative acceptor atoms of the terminal substituents^31,34^, giving rise to supramolecular arrays. The current study of intermolecular hydrogen bond interactions in solid-state symmetrically disubstituted 1,3-diaryltriazenes shows the crystal structure of compound **1** (Fig. 3) based on single-crystal X-ray structural analysis.

The crystal and other experimental data are listed in Table S2. The structure was solved by direct methods using *SIR*2004^67^. The nonhydrogen atoms were refined anisotropically by the full-matrix least-squares method using *SHELXL*97^68^. The crystal structure projections were performed using ORTEP^69^.

Attempts to locate H13 from a difference map and refinement with an isotropic displacement parameter resulted in an inconsistent value *U*_eq_ = 160(20). The final atomic coordinates of the non-hydrogen atoms in selected bond distances and angles are given in Table S2. Selected bond distances and angles are provided in Table S3. The molecular and crystalline structures are shown in Figs. 2 and 3.

Molecule **1** shows intramolecular N13 − H13···O6 hydrogen bonds related to an axial 2_1_ screw axis generating an infinite one-dimensional linear chain along the [010] direction via non-classical N13–H···O1s intermolecular interactions (Table S4).

The phenyl rings C1−C6 [r.m.s. deviation 0.0062(1) Å], slight deviation to C1 [0.0099 (1) Å], C7−C12 [r.m.s. deviation 0.0068(1) Å], and slight deviation to C8 [0.0096 (1) Å] were planar within experimental error and made an interplanar angle of 13.25° (dihedral angle between planes) demonstrating that the whole molecule is near planar.

All complementary structural characterization analyses of compound **1** agree with the data obtained by single-crystal X-ray diffraction.

### Biological activity

#### Agar diffusion antimicrobial activity test

The test compounds (alcoholic solutions) evaluate in this workare referred to as 1,3-*bis*(2-methoxy-4-nitrophenyl)triazene (**T1;** 537 µg/mL),1,3-*bis*(2-methoxy-4-nitrophenyl)triazene (**T2**; 490 µg/mL), 1,3-*bis*(2-methoxy-5-nitrophenyl)triazene **(T3**, and 1,3-*bis*(5-methoxy-2-nitrophenyl)triazene **(T4)**.

Antimicrobial sensitivity tests using the agar diffusion technique showed that compound **T1** presented an inhibition zone for all microorganisms, with the largest zone being 4.5 mm in diameter for *Salmonella* Enteritidis ATCC 13076 (Table 3).

**Table 3:** Compound 1 crystal data and structure refinement.

Unit cell dimensions	*a* = 6.2985(6) Å
	*b* = 13.1101(13) Å *β* = 94.612(5)°
	*c* = 21.4570(18) Å
Volume	1766.1(3) Å^3^
Space group	P2_1_/*c*
*Z*	4
Density (calculated)	1.442 mg.m^-3^
Absorption coefficient	0.114 mm^-1^
*F*(000)	800
Crystal size	0.09 × 0.14 × 0.33 mm^3^
Theta range for data collection	2.458 to 25.495°
Index ranges	− 7 ≤ h ≤ 7, − 15 ≤ k ≤ 15, − 25 ≤ l ≤ 25
Reflections collected	17,023
Independent reflections	3285 [*R*_int_ = 0.0289]
Completeness to theta = 25.242°	99.9%
Data/restraints/parameters	3285/0/262
Goodness-of-fit on *F*^2^	1.047
Final *R* indices [*I* > 2 sigma(I)]	*R*_1_ = 0.0384, w*R*_2_ = 0.1005
*R* indices (all data)	*R*_1_ = 0.0480, w*R*_2_ = 0.1074
Largest diff. peak and hole	0.339 and − 0.338 e Å^−3^

**Table 3:** Inhibitory activity of the triazene compounds against Gram-positive and -negative bacteria through the agar diffusion test using impregnated discs.

Microorganisms	**T1**	**T2**	**T3**	**T4**	**C**_**2**_	**C**^**+**^
*Staphylococcus aureus* ATCC 25923	3.0 ± 0.5 mm	3.0 ± 0.5 mm	4.0 ± 1.0 mm	2.0 ± 0.5 mm	R	4.5 ± 1.0 mm
*Streptococcus pyogenes* ATCC 19615	3.5 ± 0.5 mm	4.0 ± 1.0 mm	R	R	R	7.5 ± 1.0 mm
*Salmonella* Enteretidis ATCC 13076	4.5 ± 1.0 mm	3.0 ± 0.5 mm	R	R	R	10.5 ± 1.5 mm
*Escherichia coli ATCC* 29522	4.5 ± 0.5 mm	3.0 ± 0.0 mm	3.0 ± 1.0 mm	R	R	8.5 ± 1.0 mm
*Proteus mirabilis* ATCC 7002	1.0 ± 0.5 mm	R	R	3.0 ± 0.5 mm	R	10.0 ± 0.0 mm
*Pseudomonas aeruginosa* ATCC 27853	3.0 ± 0.5 mm	R	R	2.5 ± 0.5 mm	R	10.5 ± 1.5 mm
*Acinetobacter baumannii* ATCC 19606	2.0 ± 0.5 mm	1.5 ± 0.0 mm	R	R	R	5.0 ± 0.0 mm

#### Determination of the MIC and CBM/CFM of the selected species

The MIC of the study compounds determined using the microdilution method, showed antibacterial potential against all tested microorganisms, especially *Staphylococcus hemolyticus* IC 13084879, *Escherichia coli* ATCC 25922, and *Salmonella* Enteritidis ATCC 13076 that were all at 2.4 µg/mL concentration (Table 4).

**Table 4:** MIC of the four triazene compounds against Gram-positive and Gram-negative bacteria.

Microorganisms	**T1** 537 (µg/mL)	**T2** 490 (µg/mL)	**T3** 79.5 (µg/mL)	**T4** 975 (µg/mL)
*Staphylococcus aureus* ATCC 25923	5.3	4.9	7.9	9.0
*Staphylococcus hemolyticus* IC 13084879	2.6	2.4	3.9	4.0
*Streptococcus pyogenes* ATCC 19615	5.3	4.9	7.9	9.0
*Pseudomonas aeruginosa* ATCC 27853	5.3	4.9	7.9	9.0
*Escherichia coli* ATCC 25922	2.6	2.4	3.9	24.0
*Salmonella E*nteritidis *ATCC* 13076	2.6	2.4	3.9	4.0
*Proteus mirabilis* ATCC 7002	5.3	4.9	7.9	9.0
*Enterobacter cloacea* IC 12330175	5.3	4.9	7.9	9.0
*Acinetobacter baumannii* ATCC 19606	10.7	9.8	15.8	18.0 µg/mL

Studies by Silva et al.^70^ and Paraginski et al.^35^ corroborate the results of our study by showing that triazene exhibit antimicrobial activity against all tested Gram-positive and Gram-negative bacteria. In the study by Paraginski et al.^35^, the majority of Gram-negative strains had significant resistance mechanisms that may have decreased percentage activity compared to gram-positive bacteria. However, it is of great importance and interest to study new compounds against bacteria that developed resistance such as ESBL, MBL, and *AmpC* gene-carrying strains. Our four triazene compounds were effective against all Gram-negative bacteria tested in this work.

MICs determined using the microdilution method showed the antifungal potential of all four compounds against all tested microorganisms; **T1**, **T2**, **T4**, and especially **T3** had a lower MIC against *Candida albicans* ATCC 90028, *C. parapsilosis* ATCC 22019, and *C. tropicalis* IC than the starting concentration of 9.937 mg/L (Table 5).

**Table 5:** MIC of triazene compounds against ATCC standard yeasts and clinical isolates (CI).

Triazene compounds	*C. albicans *ATCC 90028 (µg/mL)	*C. parapsilosis* ATCC 22019 (µg/mL)	*C. krusei *ATCC 6258 (µg/mL)	*C. tropicalis* IC (µg/mL)
T1 537	67.125	67.125	33.562	67.125
T2 490	30.625	30.625	30.625	61.250
T3 79.5	9.937	9.937	19.875	9.937
T4 975	12.187	12.187	12.187	24.375

**T1** had the highest MIC (67.125 µg/mL) against *Candida* spp. It is important to highlight that all the tested triazene compounds (**T1** to **T4**) were effective against the standard ATCC *Candida* spp. and clinical isolates^71^. According to Paraginski et al.^35^, the antibacterial activity of triazene compounds has barely been explored, with only a few publications showing their activity against bacteria and fungi. An earlier study by Goswami and Purohit^72^ evaluated the activity of a series of hydroxytriazenes against bacteria and fungi; some showed antifungal activity and support the activity of triazenes against yeast seen in the present study and also confirming Goswami and Purohit’s observation that carboxyphenyl-containing hydroxytriazenes have better antibacterial and antifungal activities.

#### Antifungal activity against filamentous fungi determined by the poisoned food method

**Figure 4:**
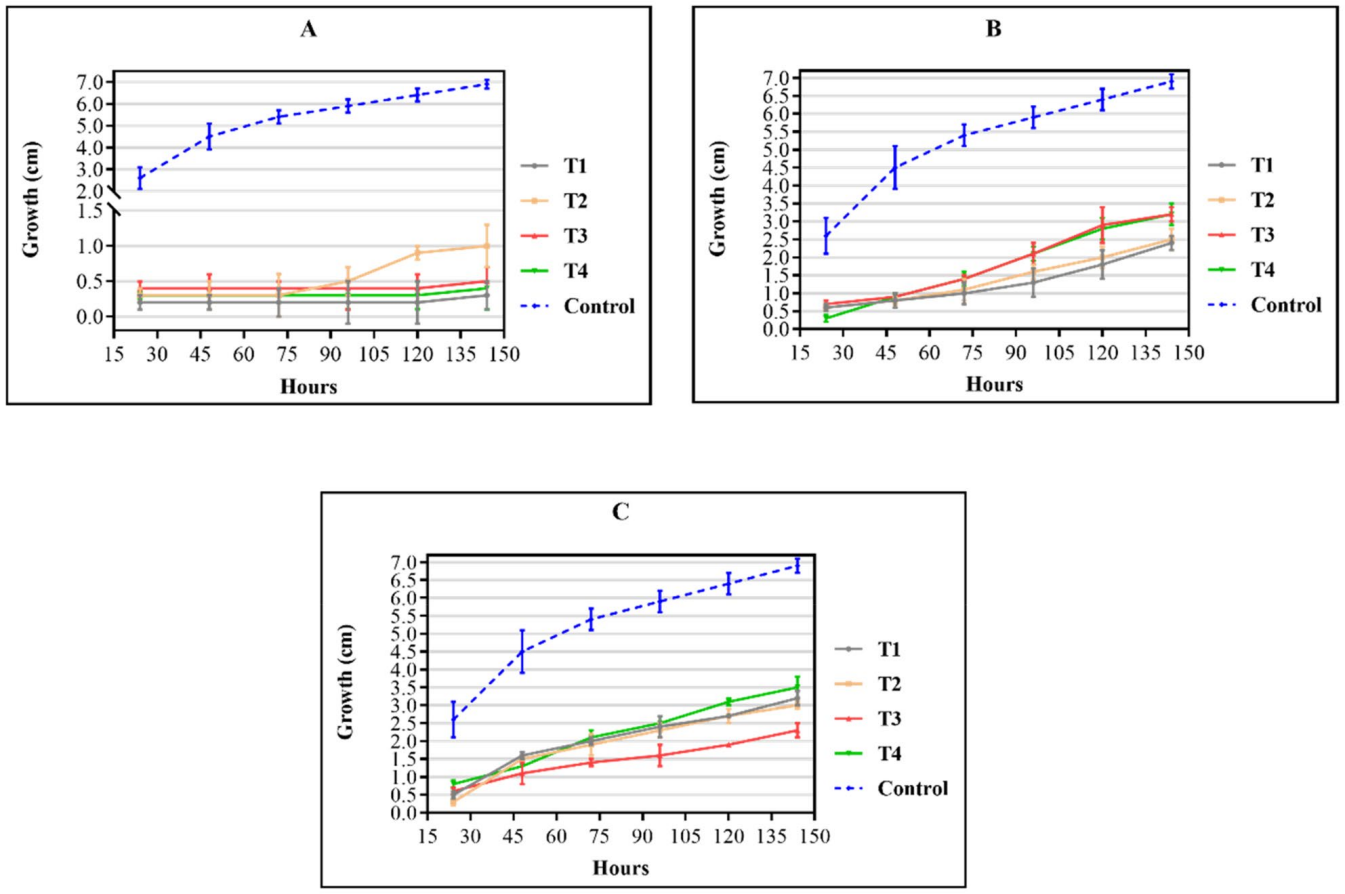
Antifungal activity of three different concentrations of the compounds against *Fusarium oxysporum* determined using the poisoned food method. (**A**) 10% concentration, (**B**) 7% concentration, and (**C**) 5% concentration. Control: the growth of filamentous fungi in a medium without drugs. Values are means (n = 3) ± standard deviations.

Figure 4 shows antifungal activity against *Fusarium oxysporum* of the triazene compounds at concentrations of 10% (A), 7% (B), and 5% (C).

**Figure 5:**
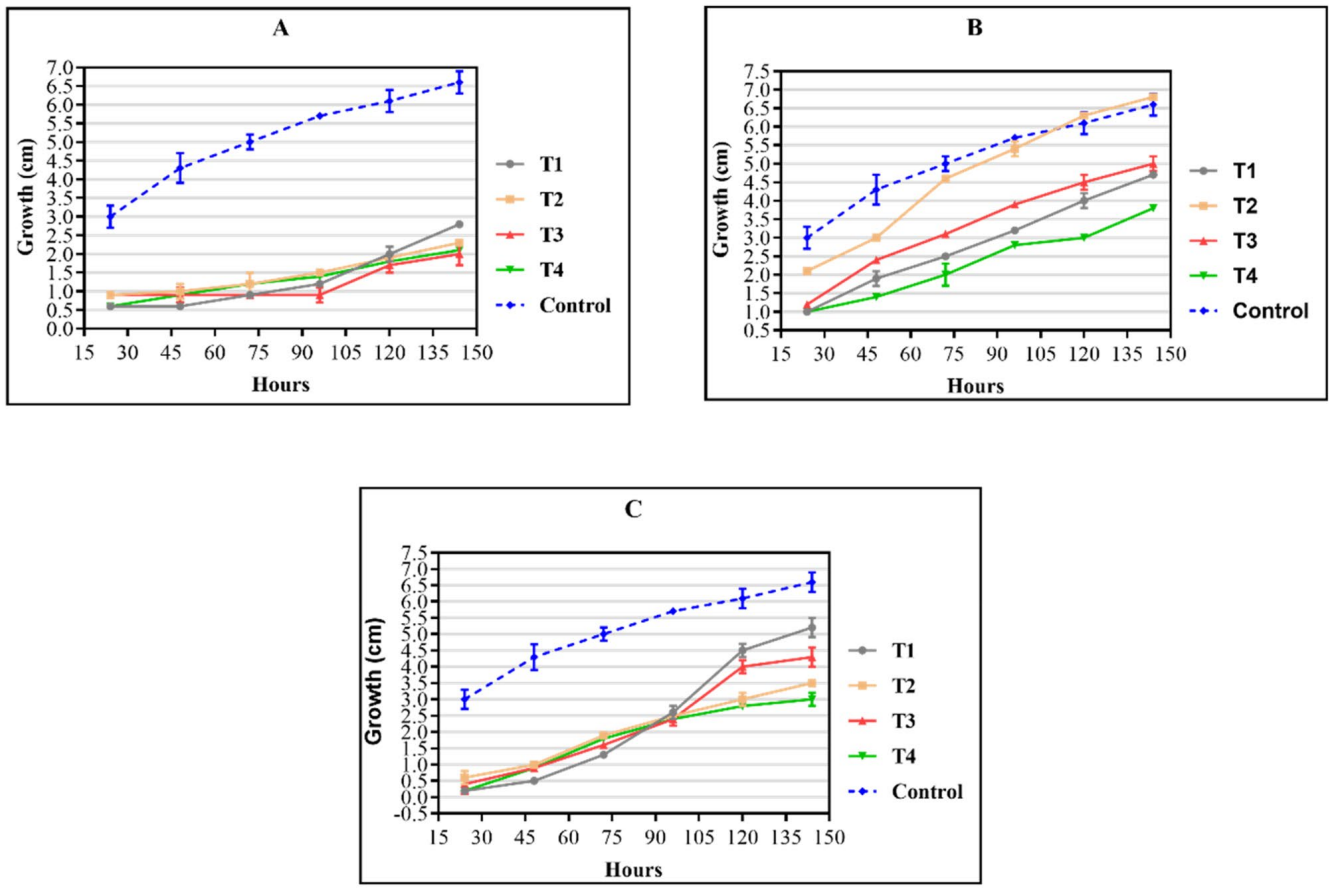
Antifungal activity of the compounds against *Penicillium janthinellum* at three different concentrations determined using the poisoned food method. (**A**) 10% concentration, (**B**) 7% concentration, and (**C**) 5% concentration. Control: the growth of filamentous fungi in a medium without drugs. Values are means (n = 3) ± standard deviations.

Figure 5 shows antifungal activity against *Penicillium janthinellum* of the triazene compounds at concentrations 10% (A), 7% (B), and 5% (C).

Compounds at 10% concentration showed a significant decrease in fungal growth (Fig. 4A and 5A); however, the fungal growth increased with decreasing concentration indicating that the activity of triazenes against filamentous fungal strains may be dose-dependent. As observed by Paraginski et al.^35^, the action mechanisms of triazene compounds against yeast and filamentous fungi remain poorly understood. The proposed triazenes’ mechanism of action involves the chelation of metal ions present in the microorganism cell wall that hinders cell wall synthesis and results in bacteriostatic activity.

#### Determination of the antibiotic effect

The analysis was performed using the lowest concentration of triazene compounds inhibiting the bacteria *Staphylococcus hemolyticus* IC 13084879, *Escherichia coli* ATCC 25922, and *Salmonella* Enteritidis ATCC 13076.

Bacteria inoculated with chloramphenicol (20 µg/mL), a known antibacterial agent, at the same quantities and volumes as in the MIC determination test, showed that *Staphylococcus hemolyticus* IC 13084879 and *Escherichia coli* ATCC 25922 growth was inhibited at concentrations greater than 0.15 µg/mL and *Salmonella* Enteritidis ATCC 13076 at concentrations greater than 0.31 µg/mL (Table 6).

**Table 6:** MIC of chloramphenicol, with the initial concentration of 20 µg/mL.

Microorganisms	MIC_c_* (µg/mL)
*Staphylococcus hemolyticus* IC 13084879	0.15
*Escherichia coli* ATCC 25922	0.15
*Salmonella* Enteritidis ATCC 13076	0.31

*MICc = Minimum inhibitory concentration of chloramphenicol.

Data in Table 7 show that chloramphenicol, the gold standard for testing antimicrobial activity, was more potent than all the tested triazenes. Chloramphenicol was 17.33-times more potent than **T1** against *Staphylococcus hemolyticus* IC 13084879 and *Escherichia coli* ATCC 25922 and 26-times more potency than **T4** against *Staphylococcus hemolyticus* IC 13084879 and *Escherichia coli* ATCC 25922.

**Table 7:** Determination of potency of triazene compounds in comparison to the activity of the known antibiotic chloramphenicol [20 µg/mL].

Microorganisms	MIC T1 (µg/mL)	MIC_c_ (µg/mL)	P_T1 (µg/mL)_	%
*Staphylococcus hemolyticus* IC 13084879	2.6	0.15	17.33	1733
*Escherichia coli* ATCC 25922	2.6	0.15	17.33	1733
*Salmonella* Enteritidis ATCC 13076	2.6	0.31	8.38	838

Microorganisms	MIC T2 (µg/mL)	MIC_c_ (µg/mL)	P_T2 (µg/mL)_	%
*Staphylococcus hemolyticus* IC 13084879	2.4	0.15	16.00	1600
*Escherichia coli* ATCC 25922	2.4	0.15	16.00	1600
*Salmonella* Enteritidis ATCC 13076	2.4	0.31	7.74	774

Microorganisms	MIC T3 (µg/mL)	MIC_c_ (µg/mL)	P_T3 (µg/mL)_	%
*Staphylococcus hemolyticus* IC 13084879	3.9	0.15	26.00	2600
*Escherichia coli* ATCC 25922	3.9	0.15	26.00	2600
*Salmonella* Enteritidis ATCC 13076	3.9	0.31	12.58	1258

Microorganisms	MIC T4 (µg/mL)	MIC_**c**_ (µg/mL)	P_**T4**_ (µg/mL)	%
*Staphylococcus hemolyticus* IC 13084879	4.0	0.15	26.00	2600
*Escherichia coli* ATCC 25922	4.0	0.15	26.00	2600
*Salmonella* Enteritidis ATCC 13076	4.0	0.31	13.00	1300

**Figure 6:**
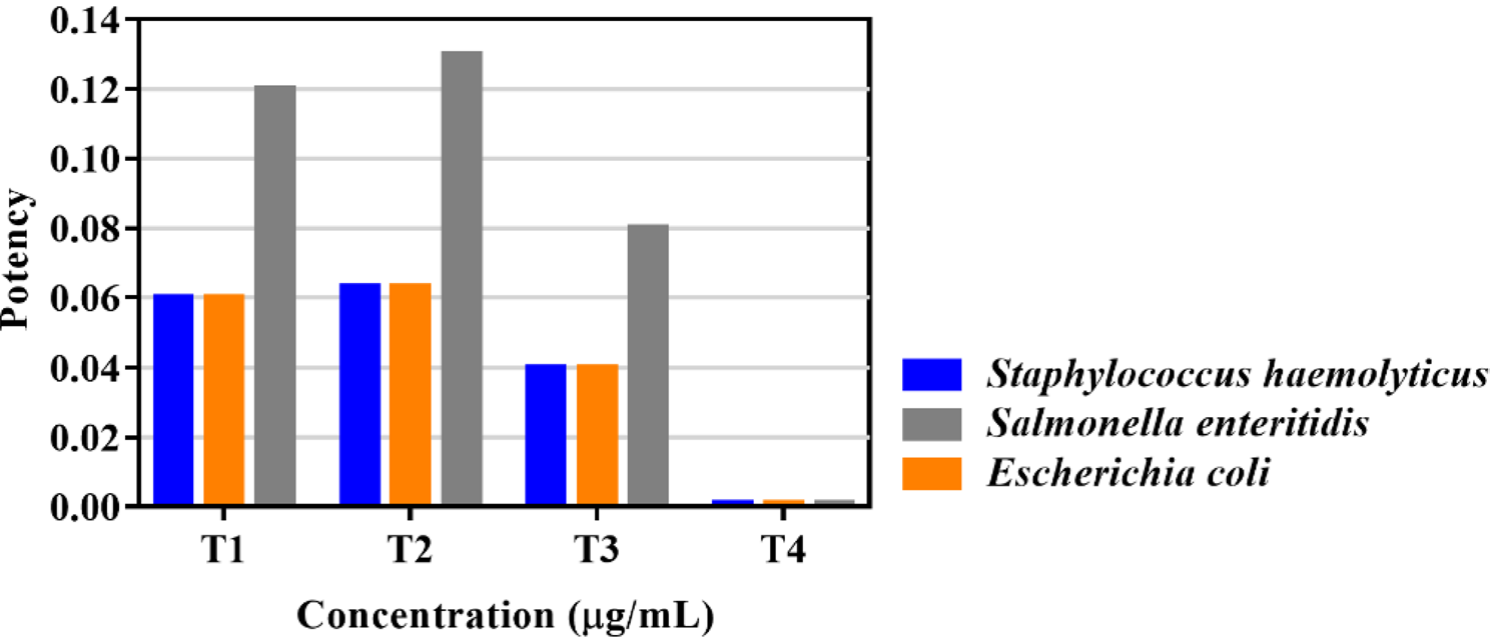
The potency of compounds **T1**, **T2**, **T3**, and **T4** against *Staphylococcus hemolyticus* IC 13084879, *Escherichia coli* ATCC 25922, and *Salmonella* Enteritidis ATCC 13076.

The compounds showed similar potency relative to chloramphenicol except for **T4** that was the least potent and significantly different from the other three tested molecules (Fig. 6). **T2** is the most potent triazene against the tested microorganisms.

Chloramphenicol was more potent than all the triazene compounds used in this research; it is a classic broad-spectrum antibiotic produced by several species of bacterial genera *Streptomyces* including *Staphylococcus venezuelae*. This antimicrobial agent is unique among natural compounds; it contains nitrobenzene connected to a propanol group and an amino group connected to a dichloroacetic acid derivative^73^.

The main mechanism of action of chloramphenicol in various bacteria involves inhibition of protein synthesis in sensitive strains; it rapidly penetrates bacterial cells, probably through facilitated diffusion. It reversibly binds to a receptor site on the 50S subunit of the bacterial ribosome^51^.

#### Assessment of mucous membrane irritation potential

Statistical analysis of the triplicate spectrophotometric results (Table 8) relating 50% hemolysis (H_50_) concentration and Denaturation Index (DI) characterized the triazene compounds as severe irritants.

**Table 8:** Mucous membrane irritation potential of triazene against sheep erythrocytes.

Triazene compounds	H_50_ (mg/mL)	DI (%)	H_50_/DI	Irritability
T1	0.33	0.4	0.90	SI
	0.33	0.4	0.90	SI
	0.33	0.4	0.90	SI
T2	0.53	0.6	0.88	SI
	0.53	0.6	0.88	SI
	0.53	0.6	0.88	SI
T3	0.49	0.5	0.98	SI
	0.49	0.5	0.98	SI
	0.49	0.5	0.98	SI
T4	0.32	0.4	0.80	SI
	0.32	0.4	0.80	SI
	0.32	0.4	0.80	SI

*SI* Severe irritant, *DI* Denaturation index, *H*_*50*_ Hemolysis 50%.

**T1**, **T2**, **T3**, and **T4** were classified as severe mucous membrane irritants, with a strong hemolysis index and cellular toxicity observed later in this study. The cytotoxicity of the compounds may be related to their hemolytic action and mucous membrane irritation potentials^9^.

### Cytotoxic assay by MTT

**Figure 7:**
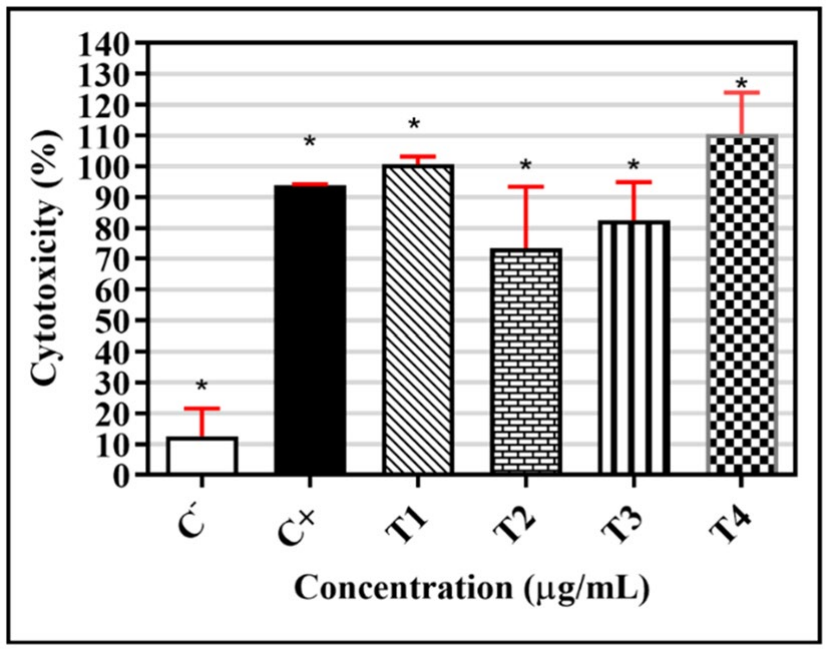
**T1**, **T2**, **T3**, and **T4** toxicity in human macrophages Cells (1.0 × 10^6^/mL) were treated with 47 µg/mL of **T1**, 530 µg/mL of **T2**, 9 µg/mL of **T3,** and 121 µg/mL of **T4** for 24 h at 37 °C and 5% CO_2_ oven. The cytotoxicity was evaluated via incubation with MTT (5 mg/mL) for 3 h at 37 °C, followed by colorimetric stabilization with SDS (10%) overnight, and subsequent absorbance reading at 540 nm. The data are expressed as mean ± standard deviation of the percentage cytotoxicity of four individual triplicates; *p < 0.05, for Mann–Whitney test, compared to the negative control.

The results showed that the triazene compounds were cytotoxic; the results were statistically different compared to the negative control (Fig. 7). After 24-h treatment, **T1** showed 100% toxicity at a concentration of 47 µg/mL; **T2**, 73.45% at 530 µg/mL; **T3**, 82.47% at 9 µg/mL; and **T4**, 100% at 121 µg/mL.

Exposure to the triazene compounds showed a significant increase in toxicity for all concentrations and C^+^ compared to the negative control, according to the one-way ANOVA test. This suggests that there is no difference between the use of **T1**, **T2**, **T3**, and **T4**.

**Figure 8:**
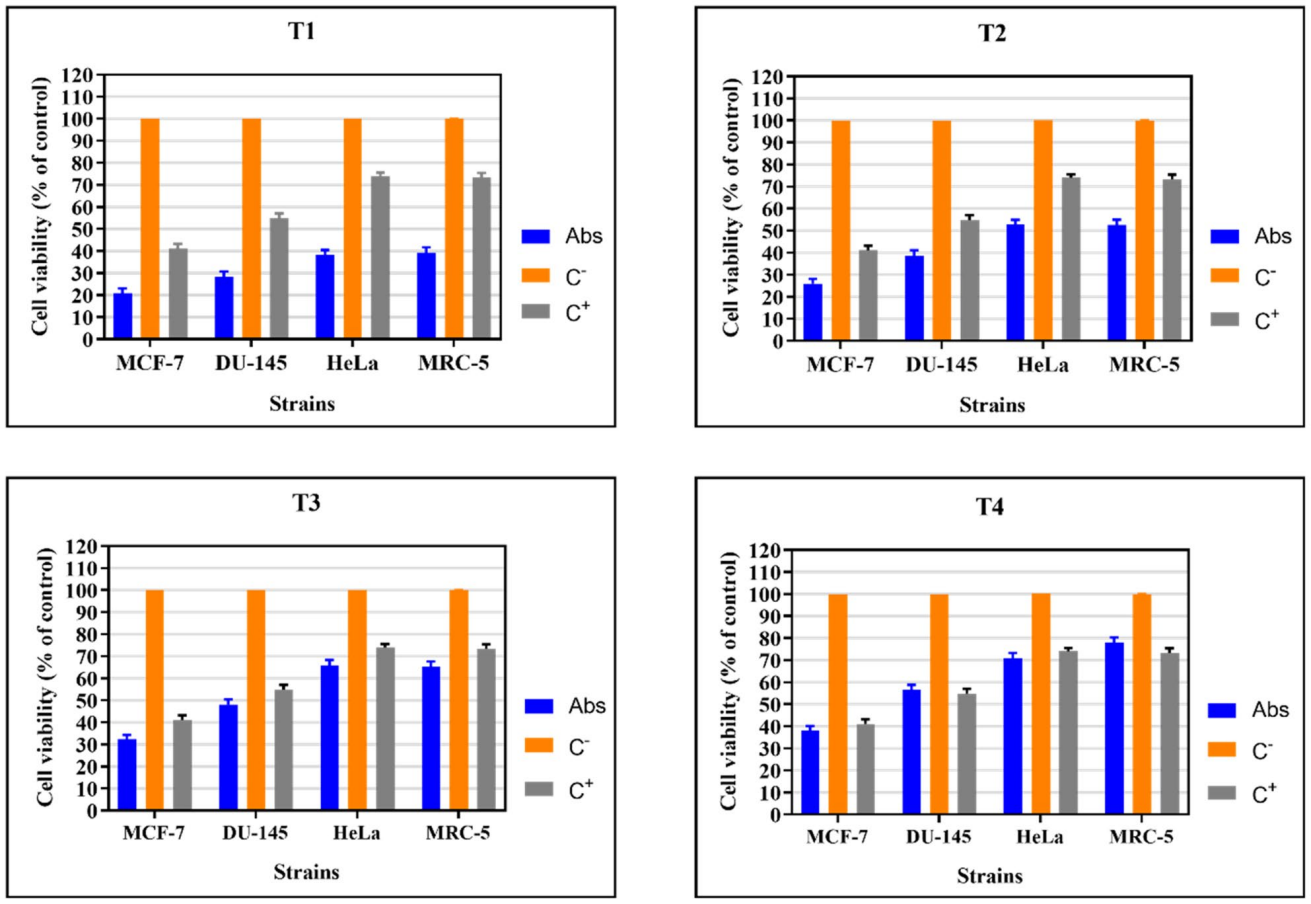
Triazene compounds **T1**, **T2**, **T3**, and **T4**. MCF-7: Human breast cancer cell line; DU-145: Prostate cancer cell line; HeLa: Malignant tumor cell line of the uterus; MRC-5: Normal cell line; C^-^: Negative control (DMEM medium); C^+^: Positive control (Triton X). Cells (1.0 × 10^6^/mL) were treated with 47 µg/mL of **T1**, 530 µg/mL of **T2**, 9 µg/mL of **T3,** and 121 µg/mL of **T4** for 24 h at 37 °C and 5% CO_2_. The cytotoxicity was evaluated via incubation with MTT (5 mg/mL) for 3 h at 37 °C, followed by colorimetric stabilization with SDS (10%) overnight, and subsequent absorbance reading at 540 nm.

All the tested triazene compounds were cytotoxic, with no significant difference compared to the positive control (Fig. 8). According to Cytarska^48^, triazenes and alkylating compounds cause changes in the DNA chain, preventing its replication^12,72^. Thus, the cytotoxic and antimicrobial activities can be attributed to the toxic potential of the triazene compounds.

**T1** and **T4** showed 100% cytotoxicity against macrophages and cells with a lower H_50_ concentration. Thus, **T1** and **T4** were the most cytotoxic in this study. Domingues et al.^9^ showed that most antineoplastic agents act on DNA or its precursors, inhibiting synthesis or causing irreparable damage. Some examples are alkylating agents, including cisplatin, and antibiotics with antitumor activity able to act in various cell cycle phases (i.e., the activity is not specific to cell phases).

Figure 7 shows the triazene compounds’ effect on reducing the viability of tumor and normal cells. Triazene compounds significantly decreased tumor cell viability. **T1** reduced cell viability to less than 40% that was greater than that of the positive control. The cytotoxic effects of alkylating compounds are related to promoting DNA alkylation^74^, preventing tumor cell multiplication and protein synthesis in these cells. The most frequent alkylation site is guanine N7; however, the mechanism of action is related to O6-guanine methylation^48,74,75^.

### Ecotoxicity test with *Danio rerio*

The ecotoxicity study suggested that the triazene compounds **T1** and **T4** were considered more toxic as they caused 100% toxicity to fish in a 15-min time interval (Table 9). Thus, we can correlate their biological activity (cytotoxic and antimicrobial) with the potential for environmental toxicity.

**Table 9:** Ecotoxicity test results; concentrations of the triazene compounds used in the fish test, MIC, and toxicity classification.

Triazene compounds	Concentration (mg/mL)	MIC (mg/mL)	Toxicity (time)
T1	0.863	0.2697	Toxic to 100% of fish (15 min)
T2	0.785	0.0024	Non-toxic to 100% of fish (48 h)
T4	0.975	0.0305	Toxic to 100% of fish (15 min)

Toxicity parameter: Caused fish death.

Non-Toxic: Did not kill the fish.

MIC: Minimum inhibitory concentration.

In a study by Paraginski et al.^35^, the tested triazene compounds showed toxicity about 10-times greater than dacarbazine, an alkylating agent used clinically as an antitumor agent. During their study, triazene compounds that showed the greatest ecotoxicity were those with the best antimicrobial activity; this corroborates the results presented in this study. **T1** and **T4** showed greater toxicity and their best activity against the standard *Candida spp* strains. This indicates that antimicrobial activity may be related to the acute toxicity of the compounds.

Paraginski et al.^35^ presented experimental data showing a correlation between the mean lethal concentration (LC50) of *Artemia salina* and the mean effective dose (ED50) obtained for tumor cell lines (cytotoxicity). Thus, it is also possible to correlate **T1** and **T4** cytotoxicity with the high death rate in the *Danio rerio* species test.

## Conclusion

The study describes the synthesis of 1,3-diaryltriazene-derived triazene compounds **1**, **2**, and **3**.

Spectroscopic and structural characterization analyses showed the structures of compounds **1**, **2**, and **3**. Moreover, the molecular structure of **1** was characterized by single-crystal X-ray diffraction. All structural characterization analyses for compounds **1**, **2**, and **3** agree with each other and with the literature.

Bacterial resistance is a major problem; few antimicrobial agents are effective in treating infections from multidrug-resistant pathogens, and a limited number of antimicrobial agents are in the final development stage. The characteristics of our compounds are promising for the potential clinical utility of these triazenes as novel antimicrobials. Our data suggest that 1,3-diaryltriazenes could represent a new class of antibacterial drugs.

The application of our triazene compounds for coordinated actions focuses on their antifungal, cytotoxic, and antibacterial activities against *Danio rerio*. The triazene compounds in this study showed significant antifungal and antibacterial activities (Gram-positive and Gram-negative) compared to triazenes reported in other studies, showing a greater range of lower MIC values. This is the first report of triazene compound activity against yeast and filamentous fungi. This evidence may be of a great impact because of the prevalence of resistance mechanisms among these microorganisms.

The most surprising result was obtained for **T3**, with a more effective MIC of 9.937 µg/mL and antifungal activity against *Candida albicans* ATCC 90,028, *C. parapsilosis* ATCC 2019, and *C. tropicallis* IC. The toxicity test against *Danio rerio* showed that **T3** and **T4** administration led to 100% fish death within 15 min, 100% cytotoxicity in macrophages, and a decrease in the viability of normal and tumor cells as well as in cells with low H_50_ concentrations.

Our data indicate that our new triazene compounds are highly toxic. Although antifungal activities may be related to cytotoxicity, the results are promising and may direct the synthesis of new active molecules of possible utility as disinfectants, hospital cleaner formulations, etc. Additional studies need to be carried out to evaluate the biological activities of these molecules when complexed with metals.

## Supplementary Information

Supplementary Information
