# Supplementary material for: Assessment of the biological potential of diaryltriazene-derived triazene compounds
Source: Sci Rep. 2021 Jan 28;11:2541. doi: 10.1038/s41598-021-81823-2 (PMC7844262; doi:10.1038/s41598-021-81823-2)
Supplement: Supplementary file 1 — Supplementary Information [file 41598_2021_81823_MOESM1_ESM.docx]

**Supplementary material**

**Assessment of the Biological Potential of**

**Diaryltriazene-derived Triazene Compounds**

Patricia de Maria Silva Figueiredo, José Costa Sampaio Filho, Alzirene de Jesus Sales Sodré, José Ribamar de Castro Júnior, Ingrid Santos Gonçalves, Rodrigo Vieira Blasques, Rodrigo de Souza Correa, Benedicto Augusto Vieira Lima, Larissa dos Anjos Marques, Denise Fernandes Coutinho, Ana Paula Silva de Azevedo dos Santos, Tássio Rômulo Silva Araújo Luz, Rita de Cassia Mendonça de Miranda, Julliana Ribeiro Alves dos Santos, Antonio Carlos Doriguetto, María Isabel Pividori, Manfredo Hörner, and Paulo Cesar Mendes Villis

***Correspondence:**  Electrochemistry and Biotechnology Laboratory (EBL), University of CEUMA (UNICEUMA), 65.065-470, São Luís, MA, Brazil. Telephone/Fax: +55 98 3214-4127.

**E-mail address:** paulo.villis@ceuma.br (P. C. M. Villis).

| **Table of contents** | | |
| --- | --- | --- |
| **Subject** |  | **Page** |
| Supplementary Figures ............................................................................................................................ | | **2** |
| **Figure S1.** Chromatogram from headspace of THF and ethanol solution for **1** | | **2** |
| **Figure S2.** Chromatogram from headspace of THF solution for **1** | | **3** |
| **Figure S3.** Espectro ^13^C NMR for **1** | | **4** |
| Supplementary Tables ................................................................................................................................ | | **5** |
| **Table S1.** Classification of the irritation potential determined by the quotient between the concentration that causes 50% hemolysis (H_50_) and denaturation index (ID) | | **5** |
| **Table S2.** Atomic coordinates (x 10^4^) and equivalent isotropic displacement parameters (Å^2^ x 10^3^) for **1**. U_eq_ is defined as one third of the trace of the orthogonalized *U*^ij^ tensor | | **6** |
| **Table S3.** Bond lengths [Å] and angles [°] for **1** | | **7** |
| **Table S4.** Hydrogen-bonding geometric parameters (Å, °) for **1** | | **8** |
| Supplemental Methods ............................................................................................................................... | | **11** |
| **Preparation of 1,3-*bis*(2-methoxy-4-nitrophenyl)triazene (1)** | | **11** |
| **Structural characterizations of (1)** | | **11** |
| **Preparation of 1,3-*bis*(2-methoxy-5-nitrophenyl)triazene (2)** | | **12** |
| **Structural characterizations of (2)** | | **12** |
| **Preparation of 1,3-*bis*(5-methoxy-2-nitrophenyl)triazene (3)** | | **13** |
| **Structural characterizations of (3)** | | **13** |
| Supplementary information ..................................................................................................................... | | **14** |

**Supplementary Figures**

**
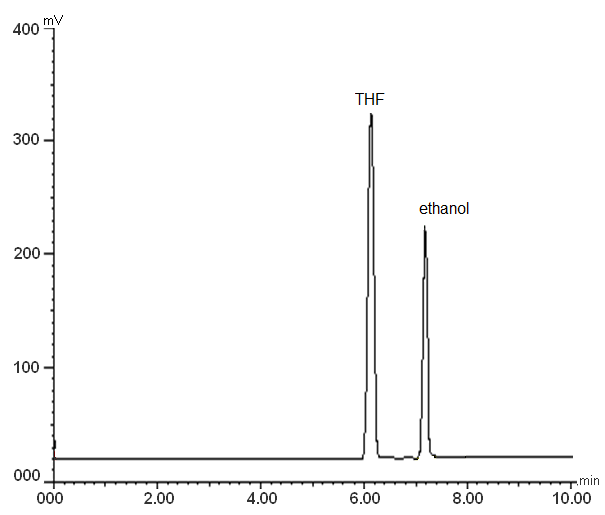
**

**Figure S1.** Chromatogram from headspace of THF and ethanol solution for **1**.

**
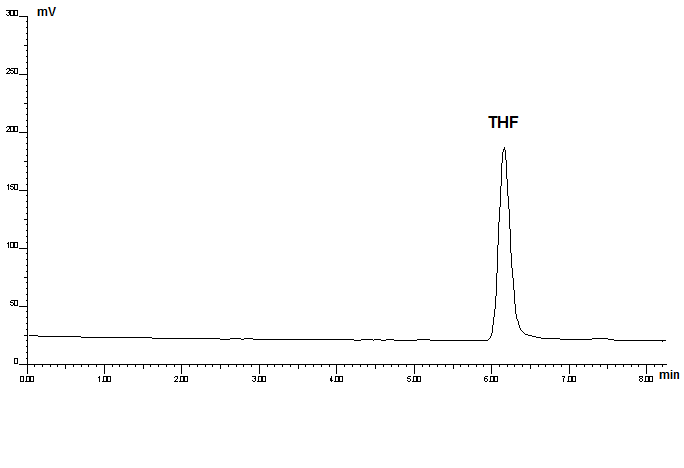
**

**Figure S2.** Chromatogram from headspace of THF solution for **1**.

**Figure S3.** Espectro ^13^C NMR for **1**.

| Supplementary Tables   \| Table S1. Classification of the irritation potential determined by the quotient between the concentration that causes 50% hemolysis (H_50_) and denaturation index (ID) \| \| \| --- \| --- \| \| Degree of Irritability \| **Irritation Type** \| \| < 0,1 \| Maximum Irritant \| \| 0,1≤ *x** ≤ 1 \| Severe irritant \| \| 1 ≤ *x ≤ 10* \| Moderate irritant \| \| 10 ≤ *x* ≤ 100 \| Mild irritant \| \| > 100 \| Non-irritating \| \| X = quotient between the concentration that causes 50% hemolysis (H_50_) and denaturation index (ID) \| \|   Table S2. Atomic coordinates (x 104) and equivalent isotropic displacement parameters (Å2 x 103) for 1. *U*_eq_ is defined as one third of the trace of the orthogonalized *U*ij tensor. | | | | |
| --- | --- | --- | --- | --- | --- | --- | --- | --- | --- | --- | --- | --- | --- | --- | --- | --- | --- | --- | --- | --- |
|  | **x** | **y** | **z** | ***U*(eq)** |
| C(1) | 6520(2) | 3265(1) | 1005(1) | 20(1) |
| C(2) | 4515(2) | 3470(1) | 679(1) | 22(1) |
| C(3) | 3743(2) | 4465(1) | 645(1) | 24(1) |
| C(4) | 4985(2) | 5241(1) | 932(1) | 22(1) |
| C(5) | 6964(2) | 5066(1) | 1243(1) | 22(1) |
| C(6) | 7725(2) | 4067(1) | 1276(1) | 20(1) |
| C(7) | 11338(2) | 799(1) | 1702(1) | 20(1) |
| C(8) | 11682(2) | -254(1) | 1832(1) | 19(1) |
| C(9) | 13571(2) | -568(1) | 2151(1) | 19(1) |
| C(10) | 15062(2) | 178(1) | 2349(1) | 19(1) |
| C(11) | 14739(2) | 1212(1) | 2237(1) | 22(1) |
| C(12) | 12872(2) | 1517(1) | 1905(1) | 23(1) |
| C(13) | 1502(3) | 2812(2) | 56(1) | 33(1) |
| C(14) | 10280(3) | -1962(1) | 1772(1) | 30(1) |
| N(1) | 4148(2) | 6296(1) | 904(1) | 27(1) |
| N(2) | 17017(2) | -147(1) | 2710(1) | 22(1) |
| N(11) | 7164(2) | 2229(1) | 1019(1) | 24(1) |
| N(12) | 8911(2) | 2048(1) | 1364(1) | 22(1) |
| N(13) | 9429(2) | 1076(1) | 1365(1) | 23(1) |
| O(1) | 2438(2) | 6450(1) | 597(1) | 40(1) |
| O(2) | 5174(2) | 6973(1) | 1192(1) | 36(1) |
| O(3) | 3481(2) | 2639(1) | 421(1) | 32(1) |
| O(4) | 17285(2) | -1067(1) | 2817(1) | 30(1) |
| O(5) | 18312(2) | 516(1) | 2901(1) | 29(1) |
| O(6) | 10055(2) | -886(1) | 1626(1) | 26(1) |
| O(1S) | 7377(4) | -97(2) | 352(1) | 37(1) |
| C(1S) | 7050(7) | 550(4) | -195(2) | 44(1) |
| C(2S) | 5239(3) | -409(2) | 503(1) | 48(1) |
| C(3S) | 6393(6) | -56(4) | -94(2) | 41(1) |

| Table S3. Bond lengths [Å] and angles [°] for 1 | |
| --- | --- |
| C(1)-C(6) | 1.395(2) |
| C(1)-N(11) | 1.418(2) |
| C(1)-C(2) | 1.420(2) |
| C(2)-O(3) | 1.3635(19) |
| C(2)-C(3) | 1.392(2) |
| C(3)-C(4) | 1.396(2) |
| C(4)-C(5) | 1.385(2) |
| C(4)-N(1) | 1.479(2) |
| C(5)-C(6) | 1.395(2) |
| C(7)-C(12) | 1.393(2) |
| C(7)-N(13) | 1.4010(19) |
| C(7)-C(8) | 1.421(2) |
| C(8)-O(6) | 1.3635(18) |
| C(8)-C(9) | 1.387(2) |
| C(9)-C(10) | 1.397(2) |
| C(10)-C(11) | 1.389(2) |
| C(10)-N(2) | 1.4648(19) |
| C(11)-C(12) | 1.384(2) |
| C(13)-O(3) | 1.4364(19) |
| C(14)-O(6) | 1.4489(19) |
| N(1)-O(1) | 1.2342(18) |
| N(1)-O(2) | 1.2347(19) |
| N(2)-O(4) | 1.2361(17) |
| N(2)-O(5) | 1.2391(17) |
| N(11)-N(12) | 1.2974(18) |
| N(12)-N(13) | 1.3158(18) |
| O(1S)-C(3S) | 1.099(4) |
| O(1S)-C(1S) | 1.450(5) |
| O(1S)-C(2S) | 1.469(3) |
| C(1S)-C(3S) | 0.929(5) |
| C(1S)-C(2S)#1 | 1.548(5) |
| C(2S)-C(3S)#1 | 1.433(5) |
| C(2S)-C(3S) | 1.592(5) |
| C(3S)-C(3S)#1 | 1.838(8) |
| C(6)-C(1)-N(11) | 124.59(13) |
| C(6)-C(1)-C(2) | 119.71(14) |
| N(11)-C(1)-C(2) | 115.69(14) |
| O(3)-C(2)-C(3) | 125.08(14) |
| Continuation Table S3 | |
| O(3)-C(2)-C(1) | 115.18(14) |
| C(3)-C(2)-C(1) | 119.73(14) |
| C(2)-C(3)-C(4) | 118.64(14) |
| C(5)-C(4)-C(3) | 122.85(15) |
| C(5)-C(4)-N(1) | 118.58(14) |
| C(3)-C(4)-N(1) | 118.57(13) |
| C(4)-C(5)-C(6) | 118.19(15) |
| C(5)-C(6)-C(1) | 120.86(14) |
| C(12)-C(7)-N(13) | 122.12(14) |
| C(12)-C(7)-C(8) | 120.26(14) |
| N(13)-C(7)-C(8) | 117.62(13) |
| O(6)-C(8)-C(9) | 124.94(14) |
| O(6)-C(8)-C(7) | 115.35(13) |
| C(9)-C(8)-C(7) | 119.71(14) |
| C(8)-C(9)-C(10) | 118.14(14) |
| C(11)-C(10)-C(9) | 123.03(14) |
| C(11)-C(10)-N(2) | 118.71(13) |
| C(9)-C(10)-N(2) | 118.22(14) |
| C(12)-C(11)-C(10) | 118.48(14) |
| C(11)-C(12)-C(7) | 120.36(14) |
| O(1)-N(1)-O(2) | 123.32(14) |
| O(1)-N(1)-C(4) | 117.87(14) |
| O(2)-N(1)-C(4) | 118.80(13) |
| O(4)-N(2)-O(5) | 123.12(13) |
| O(4)-N(2)-C(10) | 118.59(13) |
| O(5)-N(2)-C(10) | 118.29(13) |
| N(12)-N(11)-C(1) | 114.55(13) |
| N(11)-N(12)-N(13) | 112.12(13) |
| N(12)-N(13)-C(7) | 117.00(13) |
| C(2)-O(3)-C(13) | 117.43(13) |
| C(8)-O(6)-C(14) | 117.62(12) |
| C(3S)-O(1S)-C(1S) | 39.9(3) |
| C(3S)-O(1S)-C(2S) | 75.1(3) |
| C(1S)-O(1S)-C(2S) | 105.6(3) |
| C(3S)-C(1S)-O(1S) | 49.3(4) |
| C(3S)#1-C(2S)-O(1S) | 111.8(2) |
| C(3S)#1-C(2S)-C(3S) | 74.6(3) |
| O(1S)-C(2S)-C(3S) | 41.85(17) |
| Continuation Table S3 | |
| C(1S)#1-C(2S)-C(3S) | 99.3(3) |
| C(1S)-C(3S)-O(1S) | 90.8(5) |
| C(1S)-C(3S)-C(2S) | 132.7(5) |
| O(1S)-C(3S)-C(2S) | 63.1(3) |
| C(2S)#1-C(3S)-C(2S) | 105.4(3) |
| C(1S)-C(3S)-C(3S)#1 | 115.7(6) |
| O(1S)-C(3S)-C(3S)#1 | 107.1(4) |
| C(2S)#1-C(3S)-C(3S)#1 | 56.6(2) |
| C(2S)-C(3S)-C(3S)#1 | 48.7(2) |
| Symmetry transformations used to generate equivalent atoms: | |
| #1 -x+1,-y,-z | |

| Table S4. Hydrogen-bonding geometric parameters (Å,°) for 1 | | | | |
| --- | --- | --- | --- | --- |
| D−H···A | **D−H** | **H···A** | **D···A** | D−H···A |
| N13−H13···O1s | 0.86 | 2.09 | 2.881(3) | 153 |
| N13−H13···O6 | 0.86 | 2.36 | 2.6549(17) | 100 |
| (D = donor atom, A = acceptor atom) | | | | |

**Supplemental Methods**

**Preparation of 1,3-*bis*(2-methoxy-4-nitrophenyl)triazene (1)**

2-methoxy-4-nitroaniline (0.5 g, 2.97 mmol, 140–142 °C) was dissolved in 30 mL of methanol and stirred at room temperature (25 °C). Isoamyl nitrite (0. 20 mL, 1.48 mmol) was added to this solution. An orange-red reaction mixture was formed and stirred for 2 h, with the formation of a precipitate. The solvent was removed under reduced pressure and the crude product dried over P_2_O_5_ under vacuum, recrystallized from an ethanol/tetrahydrofuran (THF) mixture (1:3), and filtered.

**Chromatography and Solvent Extraction for 1**.

A carrier gas (N_2_) with a flow rate of 2 mL/min was used in the splitless mode. The retention times were 6.05 min for THF and 7.14 min for ethanol. Figure S1 show the chromatographic conditions.

The compound was transferred into appropriately sealed flasks and stored at 100°C for 1 h, then 100 µL of headspace was directly injected into the GC injection port. A peak was observed at 6.08 min in the chromatogram, corresponding to the THF molecule retention time. Figure S2 show the chromatogram and solvent extraction.

**Structural characterizations of (1):** Yield 60 % (0.35 g, 0.89 mmol) based on 2-methoxy-4-nitroaniline, mp 203–205 °C. IR (KBr pellet, cm^-1^) 3324 (ν N-H) s; 1519 (ν_as_ NO_2_) vs; 1471 (ν_s_ N=N) s; 1467 (δ NO_2_) vs; 1337 (ν_s_ NO_2_) vs; 1168 (ν_s_ N-N) s; and 863 (ν_s_ C-N) w. (IR data: vs, very strong; s, strong; and w, weak). CHNS/O Analyzer for C_16_H_19_N_5_O_7_: C, 48.85; H, 4.87; N, 17.80; and O, 28.48. Found C, 48.82; H, 4.37; N, 18.30; and O, 28.51. ^1^H NMR (300 MHz, (CDCl_3_), ppm): δ 1,82−1,75 (s, CH_3_), 2,85−2,82 (d, CH_2_), 3,66−3,59 (m, −OCH_3_), 4,10 (s, 1H, NH), 3,96−3,95 (s, 1H, OH), and 7,93−7,88 (m, 6H, ArH). ^13^C NMR (75.43 MHz, (CDCl_3_), ppm): δ 56.73 (s, C1, C2, OCH_3_), 107.27 (s, C16, C26, O_2_NC_6_H_3_), and 117.66 (s, C13, C15, C23, C25, O_2_NC_6_H_3_), as show in Figure S3. UV-vis in THF (5 x 10^-4^ mol L^-1^): n→ π* (N=N) λ_max_ 425 nm, n→ π* (NO_2_) λ 277 nm, π→σ* (N-H), and π→π* (C=CAr) λ 239 nm.

**Preparation of 1,3-*bis*(2-methoxy-5-nitrophenyl)triazene (2)**

Isoamylic nitrite (0.2 mL, 1.48 mmol) was added to 2-methoxy-5-nitroaniline (0.5 g, 2.97 mmol) at 117–119 °C and dissolved in 30 mL of ethanol at room temperature (25 °C), with continuous stirring. The yellow precipitate formed was separated via filtration and washed with small portions of cold methanol/ethanol (1:1). The solvent was removed under reduced pressure, and the resulting orange-red powder was dissolved in tetrahydrofurane/acetone (1:1) and filtered.

**Structural characterizations of (2):** Yield 58.24 % (0.34 g; 0.86 mmol) from 2-methoxy-5-nitroaniline, mp 205–208 °C. IR (KBr pellet, cm^-1^) 3328 (ν N-H) s; 1086 (ν_as_ NO_2_) vs; 1471 (ν_s_ N=N) s; 1467 (ν_s_ NO_2_) vs; and 1164 (ν_s_ N-N) s. 846 (ν_s_ C-N) w. (IR data: vs, very strong; s, strong; and w, weak). CHNS/O Analyzer for C_14_H_13_N_5_O_6_: C, 48.42; H, 3.77; N, 20.17; and O, 27,64. Found C, 48.55; H, 3.57; N, 20.01; and O, 27,87. ^1^H NMR (300 MHz, (CDCl_3_), ppm): δ3.96 (s, 6H, OCH_3_), 7.67 (d, H16, H26, O_2_NC_6_H_3_), 7.80 (s, H13, H23, O_2_NC_6_H_3_), 7.91 (d, H15, H25, O_2_NC_6_H_3_OCH_3_), and 10.14 (s, H1, NH). UV-Vis in THF (5 x 10^-4^ mol L^-1^): n→ π* (N=N) λmax. 381 nm, n→ π* (NO_2_) λ 353 nm, π→σ* (N-H), and π→π* (C=CAr) λ 300 nm.

**Preparation of 1,3-*bis*(5-methoxy-2-nitrophenyl)triazene (3)**

5-methoxy-2-nitroaniline (0.5 g, 2.97 mmol, 128 - 132 °C) was dissolved in 20 mL of ethanol/ethyl ether (2:1) and stirred at room temperature (25 °C). Isoamyl nitrite (0.20 mL, 1.48 mmol) was added to this solution. The reaction mixture was stirred for 2 h. After removing the solvent under reduced pressure, 0.35 g of the titled compound was obtained as a yellow oil.

**Structural characterizations of (3):** Yellow oil, Yield 60 % (0.35 g, 0.89 mmol) based on 5-methoxy-2-nitroaniline. IR (KBr pellet, cm^-1^) 3328 (νN-H) s; 1519 (ν_as_NO_2_) vs; 1471 (ν_s_ N=N) s; 1337 (ν_s_ NO_2_) vs; 1168 (ν_s_ N-N) s; and 850 (ν_s_ C-N) w. (IR data: vs, very strong; s, Strong; and w, weak).). ^1^H NMR (300 MHz, (CDCl_3_), ppm): δ4.05 (s, 6H, OCH_3_), 7.77 (d, H16, H26, O_2_NC_6_H_3_), 7.86 (s, H13, H23, O_2_NC_6_H_3_), 7.94 (d, H15, H25, O_2_NC_6_H_3_OCH_3_), and 10.54 (s, H1, NH). UV-Vis in THF (5 x 10^-4^ mol/L): n→ π* (N=N) λmax. 361 nm, n→ π* (NO_2_) λ 227 nm, π→σ* (N-H), π→π* (C=CAr) λ 200 nm.

**Supplementary information**

Crystallographic data for the structural analysis have been deposited in the Cambridge Crystallographic Data Centre (CCDC N. 661902). Further details on the crystal structure investigation are available free of charge via www.ccdc.cam.ac.uk/conts/retrieving.html (or from the CCDC, 12 Union Road, Cambridge CB2 1EZ, UK; fax: +44 1223 336033; e-mail: deposit@ccdc.cam.ac.uk).
